# Epidemiology and Prognosis of Intensive Care Unit–Acquired Bloodstream Infection

**DOI:** 10.4269/ajtmh.19-0877

**Published:** 2020-04-20

**Authors:** Hatem Kallel, Stephanie Houcke, Dabor Resiere, Michaella Roy, Claire Mayence, Cyrille Mathien, Joy Mootien, Magalie Demar, Didier Hommel, Felix Djossou

**Affiliations:** 1 Intensive Care Unit, Cayenne General Hospital, Cayenne, French Guiana;; 2 Intensive Care Unit, Martinique University Hospital, Fort-de-France, Martinique;; 3 Intensive Care Unit, GHRSMA, Mulhouse, France;; 4 Laboratory of Microbiology, Cayenne General Hospital, Cayenne, French Guiana;; 5 Tropical and Infectious Diseases Department, Cayenne General Hospital, Cayenne, French Guiana

## Abstract

Intensive care unit–acquired bloodstream infections (ICU-BSI) are frequent and are associated with high morbidity and mortality rates. We conducted this study to describe the epidemiology and the prognosis of ICU-BSI in our ICU and to search for factors associated with mortality at 28 days. For this, we retrospectively studied ICU-BSI in the ICU of the Cayenne General Hospital, from January 2013 to June 2019. Intensive care unit–acquired bloodstream infections were diagnosed in 9.5% of admissions (10.3 ICU-BSI/1,000 days). The median delay to the first ICU-BSI was 9 days. The ICU-BSI was primitive in 44% of cases and secondary to ventilator-acquired pneumonia in 25% of cases. The main isolated microorganisms were Enterobacteriaceae in 67.7% of patients. They were extended-spectrum beta-lactamase (ESBL) producers in 27.6% of cases. Initial antibiotic therapy was appropriate in 65.1% of cases. Factors independently associated with ESBL-producing Enterobacteriaceae (ESBL-PE) as the causative microorganism of ICU-BSI were ESBL-PE carriage before ICU-BSI (odds ratio [OR]: 7.273; 95% CI: 2.876–18.392; *P* < 0.000) and prior exposure to fluoroquinolones (OR: 4.327; 95% CI: 1.120–16.728; *P* = 0.034). The sensitivity of ESBL-PE carriage to predict ESBL-PE as the causative microorganism of ICU-BSI was 64.9% and specificity was 81.2%. Mortality at 28 days was 20.6% in the general population. Factors independently associated with mortality at day 28 from the occurrence of ICU-BSI were traumatic category of admission (OR: 0.346; 95% CI: 0.134–0.894; *P* = 0.028) and septic shock on the day of ICU-BSI (OR: 3.317; 95% CI: 1.561–7.050; *P* = 0.002). Mortality rate was independent of the causative organism.

## INTRODUCTION

Bloodstream infections (BSIs) represent the third-most commonly recorded infection in intensive care units (ICUs).^1^ They occur in approximately 5–15% of all patients within the first month of hospitalization in ICU,^2,3^ with an incidence rate between five and 19 per 1,000 patient days.^4^ They are associated with high morbidity and mortality rates and are a marker of adverse outcomes. The case fatality rate associated with BSIs is 15–20%. It rises to 35–50% in case of organ failure.^2,3,5–7,9^ In the global population, BSI accounts for 1% excess mortality, with 5% of attributed deaths in ICU.^2^ Bloodstream infections also increases the length of ICU stay and healthcare-related costs.^9^

Bloodstream infections caused by multidrug-resistant bacteria are considered as a public health problem worldwide.^5,9,10^ They represent an additional burden of disease with higher mortality, longer ICU stay, longer delay in starting effective antimicrobials, and higher costs rather than BSIs caused by susceptible bacteria.^3,5,6,11–15^

On the other hand, early and appropriate antibiotic therapy after blood cultures is an important issue in patients with intensive care unit–acquired bloodstream infections (ICU-BSIs).^9,16^ It has been shown to reduce mortality and to improve clinical outcomes, particularly in severely affected patients.^17^ However, this relationship is controversial and treatment adequacy was sometimes unrelated to outcomes.^18^

The main objective of our study is to describe the epidemiology and the impact on outcomes of ICU-BSI. The secondary objectives are to search for factors associated with ICU-BSIs caused by extended-spectrum beta-lactamase–producing Enterobacteriaceae (ESBL-PE), and the impact of ESBL-PE carriage on the incidence of ICU-BSIs caused by ESBL-PE.

## MATERIALS AND METHODS

### Setting and patients.

Our study is retrospective. It was conducted over 78 months (6.5 years, from January 2013 to June 2019) in the medical/surgical ICU of the Cayenne General Hospital, French Guiana (FG). Our hospital is a 510-bed general center that serves as a first-line medical center for an urban population of 150,000 inhabitants and as a referral center (being the only ICU in the region) for a larger population coming from the costal side of the Amazonian region (French Guiana, Brazil, and Suriname). Indeed, French Guiana is located in the Amazonian region (from Guiana to northern states of Brazil). It has borders with Brazil to the east and south through the Oyapock River, and Suriname to the west through the Maroni River. Patients living in the Brazilian and Suriname riversides consult in the FG hospitals and are then transferred to ICU in Cayenne Hospital when needed. In the field of critical care, there is no cooperation established between FG and border countries. In addition, the nearest ICU in Suriname is in Paramaribo (336 km from Cayenne) and in Brazil is in Macapa (Amapa state, 782 km from Cayenne).

Our ICU comprises eight single and three double-bed rooms with a 1:2.5 nurse-to-patient ratio. All patients have dedicated equipment for care and monitoring. Hand hygiene is based on alcohol hand rub (at room entrance and exit and between each distinct procedure of care) and the use of single-use gloves and gowns in case of close contact with patients and potential exposure to body fluids during nursing.

We included all patients hospitalized in our ICU for more than 48 hours and who had acquired BSI during their ICU stay. For each patient, only the first episode of ICU-BSI is considered. For patients who were readmitted to ICU, we studied only the first ICU admission during the same hospital stay.

Screening for multidrug-resistant bacteria (MDR-B) carriage was performed using rectal swab sampling according to the French Society of Hospital Hygiene recommendations.^19^ Patients are routinely screened on ICU admission, and then weekly during the ICU stay. Extended-spectrum beta-lactamase production was confirmed by the double-disk diffusion method using ceftazidime or cefotaxime with clavulanic acid.^20^

Blood cultures were performed using aerobic (Bact/ALERT FA plus, Biomerieux, Inc., Durham, NC) and anaerobic (Bact/ALERT FN plus, Biomerieux, Inc., Durham, NC) blood culture vials incubated in a BacT/ALERT 3D (bioMérieux, Marcy l’Etoile, France). The positive blood culture vials were subcultured on blood and chocolate PolyVitex agar plates. All isolates were then identified using MALDI-TOF mass spectrometry (MaldiBiotyper 3.0, Bruker Daltonique, Marnes la Vallée, France).

Antimicrobial susceptibility testing was carried out using the agar disk diffusion method (Bio-Rad) or an automated broth micro-dilution method (Phoenix, BD Diagnostics, Oxford, United Kingdom). The breakpoints used were those defined by the French Committee for Antimicrobial Susceptibility Testing (http://www.sfmmicrobiologie.org/UserFiles/files/casfm/CASFM%20V1_0%20FEV_2018.pdf).

## DATA COLLECTION

Medical charts were reviewed using a standardized data set to collect demographic characteristics: clinical, biological, microbiological data, and outcomes for each patient.

The following data were collected: gender, age, type of admission, Simplified Acute Physiology Score (6); organ dysfunction at admission (acute change in the total Sequential Organ Failure Assessment [SOFA] score ≥ 2 points)^21^; location before ICU admission, hospitalization, and administration of antibiotics in the previous year (stratified according to receipt within 6 months or 3 months of admission or earlier); presence of underlying diseases; exposure to central venous or arterial catheterization (CVC, AC); mechanical ventilation (MV); renal replacement therapy (RRT); antibiotics administered during hospitalization in ICU; MDR-B carriage, including ESBL-PE carriage; ICU-acquired infections; length of ICU stay; and outcome at 28 days from the diagnosis of the first episode of ICU-BSI and at discharge from ICU.

Our database has been registered at the Commission National de l'Informatique et des Libertés (registration no. 2209669), in compliance with French law on electronic data sources.

## DEFINITIONS

Infections were defined according to the CDC definitions.^22^ Intensive care unit–acquired bloodstream infection was defined by an infection onset occurring at least 48 hours after ICU admission, with one positive blood culture unrelated to an infection incubating at ICU admission. Coagulase-negative *Staphylococcus* bacteremia was defined by two blood cultures showing the same phenotype on separate occasions within a 48-hour period or at least one positive blood culture for clinical sepsis, no other infectious process, and antibacterial agent treatment initiated by the attending physician.^23^ In the absence of a known source, ICU-BSI was classified as primary. Secondary BSI was defined by the recovery of the same microorganism from one blood culture and from a suspected source.^6,24–26^ All catheter-related infections were documented by quantitative tip culture.^27^ The day of the appropriate antimicrobial therapy initiation was recorded. The primary site of infection was clinically suspected and bacteriologically documented with the same bacterial identification as that in the blood culture.

Prior antibiotic exposure in ICU was defined as the use of at least one dose of any antimicrobial treatment from admission until the day before ICU-BSI. Immunosuppression included the following: ongoing neoplasia, hemopathy, HIV infection, immune-suppressive therapy (i.e., corticotherapy > 20 mg/day, chemotherapy, or immune-suppressive treatment).

## STATISTICAL ANALYSIS

Results are reported as median and interquartile range (IQR: 1st–3rd quartiles) or numbers with percentages.

Initial bivariate statistical comparisons were conducted using the Chi-square or Fisher’s exact test for categorical data and the Mann–Whitney U test for continuous data. To compare subgroups, we used multivariable logistic regression with a backward procedure. Nonredundant variables selected by bivariate analysis (*P ≤* 0.05) and considered clinically relevant were entered into a logistic regression model. Results are expressed as odds ratios (OR) with their 95% CIs. A *P*-value ≤ 0.05 was considered statistically significant. We calculated the sensitivity (Ss), specificity (Sp), positive, and negative predictive values (PPV and NPV) to assess the diagnosis value of the tests.

All statistical analyses were carried out with Excel (2007) and IBM SPSS Statistics for Windows, version 24 (IBM Corp., Armonk, NY).

## RESULTS

During the study period, we recorded 2,353 admissions resulting in 28,627 days of hospitalization in the ICU. Among them, 223 (9.5%) developed ICU-BSI and were included in our study. The median number of ICU-BSI was 29 cases per year (IQR: 26–37). The time from ICU admission to ICU-BSI was 9 days (IQR: 5–16). The total number of days of hospitalization without ICU-BSI was 21,706 days giving a density-incidence of 10.3 ICU-BSI/1,000 days of hospitalization.

The median age of our patients was 49 years (IQR: 35–61) and 67.3% of them were men. One or more comorbidity was recorded in 57.8% of patients. The main reasons for admission were trauma (30.5%), coma (15.3%), acute respiratory failure (13.4%), and shock (13%). Epidemiological and clinical characteristics of all patients at admission to ICU are reported in Table 1.

**Table 1:** Epidemiologic and clinical characteristics of the study population

Variable	Population size	Result*
Age (years)	223	49 (35–61)
Male gender	223	150 (67.3%)
BMI	191	25 (22–30)
Simplified Acute Physiology Score	219	50 (40–64)
Comorbidities	223	129 (57.8%)
Arterial hypertension	223	71 (31.8%)
Diabetes mellitus	223	29 (13%)
Cancer	223	9 (4%)
Immunodeficiency	223	40 (17.9%)
Chronic renal failure	223	11 (4.9%)
Chronic respiratory failure	223	4 (1.8%)
Sickle cell disease	223	10 (4.5%)
Type of admission		
Medical	223	158 (70.9%)
Elective surgery	223	0
Emergent surgery	223	66 (29.1%)
Traumatic	223	68 (30.5%)
Reason for admission		
Trauma	223	68 (30.5%)
Coma	223	34 (15.3%)
Acute respiratory failure	223	30 (13.4%)
Shock	223	29 (13.0%)
Cardiac arrest	223	16 (7.2%)
Status epilepticus	223	7 (3.1%)
Intra-abdominal infection	223	6 (2.7%)
Burn	223	6 (2.7%)
Meningoencephalitis	223	5 (2.2%)
Other	223	22 (9.7%)
Antibiotics during previous year	223	27 (12.1%)
In the last 3 months	223	18 (8.1%)
In the last 3–6 months	223	5 (2.2%)
In the last 6–12 months	223	4 (1.8%)
Hospitalization during the previous year	223	44 (19.1%)
In the last 3 months	223	21 (9.4%)
In the last 3–6 months	223	6 (2.7%)
In the last 6–12 months	223	17 (7.6%)

* nb (%) or median (IQR).

Active infection at admission was recorded in 117 patients (52.5%) and associated BSI was recorded in 23 of them (19.7%). Antibiotics were prescribed in 69.1% of patients at admission to ICU.

During the ICU stay, 92.8% of patients received invasive MV, 19.3% received RRT, 96.5% had central venous, and 92.4% had arterial catheterization. Therapeutic procedures and antibiotics exposure during hospitalization in ICU are reported in Table 2.

**Table 2:** Therapeutic management during hospitalization in ICU

Variable	Nb	Result
Mechanical ventilation	223	207 (92.8%)
Time from admission to MV (days)	207	0 (0–0)
MV at admission to ICU	207	177 (79.4%)
MV more than 48 hours	207	204 (98.6%)
Duration of MV (days)	207	20 (13–33)
Overall duration of MV (days)	207	6,470
Duration of MV without VAP (days)	207	3,765
Tracheostomy	207	37 (17.9%)
Renal replacement therapy	223	43 (19.3%)
Time from admission to RRT (days)	43	1 (0–8)
Central venous catheterization	223	216 (96.5%)
Overall duration of CVC (days)	216	6,382
Duration of CVC without infection (days)	216	5,495
Arterial catheterization	223	206 (92.4%)
Overall duration of AC (days)	206	3,948
Duration of AC without infection (days)	206	3,818
ATB exposure during hospitalization	223	206 (92.4%)

AC = arterial catheterization; CVC = central venous catheter; ICU = intensive care unit; MV = mechanical ventilation; RRT = renal replacement therapy; VAP = ventilator-associated pneumonia.

One hundred forty-one patients (63.2%) were MDR-B carriers and 121 patients (54.3%) carried ESBL-PE. Twenty-seven patients (12.1%) were screened positive for ESBL-PE at admission to ICU. The main ESBL-PE isolated at admission were *Klebsiella pneumoniae* and *Escherichia coli* (40.7% and 37% of ESBL-PE carriers at admission, respectively). The rates of MDR-B carriage at admission and during ICU stay are reported in Table 3.

**Table 3:** ESBL-PE carriage at admission and during ICU stay*

	At admission, *n* (%)	ICU acquired, *n* (%)	Total, *n* (%)
*Escherichia coli*	10 (52.6)	9 (47.4)	19 (100)
*Klebsiella pneumoniae*	11 (16.2)	57 (83.8)	68 (100)
*Enterobacter cloacae*	5 (18.5)	22 (81.5)	27 (100)
*Serratia marcescens*	1 (16.7)	5 (83.3)	6 (100)
*Enterobacter aerogenes*	0	3 (100)	3 (100)
*Enterobacter asburiae*	1 (50)	1 (50)	2 (100)
*Citrobacter freundii*	0	1 (100)	1 (100)
*Klebsiella oxytoca*	0	1 (100)	1 (100)
Total	28 (22)	99 (78)	127 (100)

ESBL-PE = extended-spectrum beta-lactamase–producing Enterobacteriaceae; ICU = intensive care unit. Six patients were carrying two ESBL-PE (one at admission and five during ICU stay).

* Only the first ESBL-PE carriage in each patient was reported.

During ICU stay, we recorded 453 episodes of intensive care unit-acquired infection (ICU-AI). Of these, 64 episodes were recorded before, and 166 after the studied episode of ICU-BSI. The median duration of hospitalization without ICU-BSI was 9 days (IQR: 5–16). The studied ICU-BSI was primary in 98 patients (44%) and secondary to an identified source in 125 cases (56%) (Table 4).

**Table 4:** Intensive care unit-AI before and after the studied episode of ICU-BSI

Site of ICU-AI	ICU-AI before the first ICU-BSI, *n* (%)	The first ICU-BSI,*n (%)	ICU-AI after the first ICU-BSI, *n* (%)		Total, *n* (%)
			Without BSI	With BSI	
Primary BSI	0	98 (62.4)	0	59 (37.6)	157 (100)
Ventilator-associated pneumonia	42 (29.8)	57 (40.4)	34 (24.1)	8 (5.7)	141 (100)
Catheter-related	9 (10,3)	39 (44.8)	15 (17.2)	24 (27.6)	87 (100)
Urine	5 (17,2)	15 (51.7)	6 (20.7)	3 (10.3)	29 (100)
Cutaneous	2 (20)	5 (50)	1 (10)	2 (20)	10 (100)
Pulmonary	2 (20)	4 (40)	3 (30)	1 (10)	10 (100)
Abdominal	0	2 (40)	2 (40)	1 (20)	5 (100)
Endocardial	0	2 (100)	0	0	2 (100)
Surgical site infection	0	1 (50)	0	1 (50)	2 (100)
Bronchitis	1 (50)	0	1 (50)	0	2 (100)
Gynecologic	1 (100)	0	0	0	1 (100)
Ophthalmic	1 (100)	0	0	0	1 (100)
Bone	1 (33,3)	0	2 (66.7)	0	3 (100)
Sinusitis	0	0	1 (100)	0	1 (100)
Neuromeningeal	0	0	2 (100)	0	2 (100)
Total	64	223	67	99	453

ICU = intensive care unit; ICU-BSI = intensive care unit–acquired bloodstream infection.

* The studied episode.

Intensive care unit–acquired bloodstream infection was caused by one micro-organism in 184 cases (82.5%) and two microorganisms in 39 cases (17.5%). The microorganism recovered was an Enterobacteriaceae in 151 patients (67.7%). It was an ESBL producer in 37 cases (24.5%). *Candida* spp. caused 10 cases (4.5%) of ICU-BSI. In two cases, there was coinfection with *Candida* spp. and *K. pneumoniae*. The responsible microorganisms recovered according to the site of ICU-BSI are reported in Table 5.

**Table 5:** Responsible microorganisms according to the site of intensive care unit–acquired bloodstream infections

	Primary bloodstream infection, *n* (%)	Ventilator-associated pneumonia, *n* (%)	Catheter-related bloodstream infection, *n* (%)	Urinary, *n* (%)	Other, *n* (%)	Total, *n* (%)
Gram-positive cocci	25 (22.1)	7 (10)	17 (37.8)	4 (21.1)	4 (26.7)	57 (21.8)
* Staphylococcus aureus*	3 (2.7)	7 (10)	12 (26.7)	2 (10.5)	1 (6.7)	25 (9.5)
* *Coagulase-negative staphylococci	12 (10.6)	0	2 (4.4)	0	2 (13.3)	16 (6.1)
* Enterococcus faecalis*	6 (5.3)	0	2 (4.4)	1 (5.3)	1 (6.7)	10 (3.8)
* Streptococcus* spp.	3 (2.7)	0	1 (2.2)	1 (5.3)	0	5 (1.9)
* Enterococcus faecium*	1 (0.9)	0	0	0	0	1 (0.4)
Gram-negative bacilli	81 (71.7)	62 (88.6)	26 (57.8)	13 (68.4)	11 (73.3)	193 (73.7)
* *Enterobacteriaceae	63 (55.8)	48 (68.6)	18 (40)	13 (68.4)	9 (60)	151 (57.6)
* Klebsiella pneumoniae*	21 (18.6)	24 (34.3)	9 (20)	8 (42.1)	3 (20)	65 (24.8)
* Enterobacter cloacae*	14 (12.4)	14 (20)	2 (4.4)	0	2 (13.3)	32 (12.2)
* Escherichia coli*	8 (7.1)	1 (1.4)	0	4 (21.1)	3 (20)	16 (6.1)
* Enterobacter aerogenes*	8 (7.1)	1 (1.4)	4 (8.9)	1 (5.3)	0	14 (5.3)
* Serratia marcescens*	3 (2.7)	5 (7.1)	1 (2.2)	0	0	9 (3.4)
* Citrobacter koseri*	3 (2.7)	2 (2.9)	1 (2.2)	0	0	6 (2.3)
* Enterobacter asburiae*	1 (0.9)	0	1 (2.2)	0	0	2 (0.8)
* Klebsiella varicola*	1 (0.9)	1 (1.4)	0	0	0	2 (0.8)
* Citrobacter freundii*	1 (0.9)	0	0	0	0	1 (0.4)
* Citrobacter yougae*	1 (0.9)	0	0	0	0	1 (0.4)
* Morganella morganii*	0	0	0	0	1 (6.7)	1 (0.4)
* Pantoea dispersa*	1 (0.9)	0	0	0	0	1 (0.4)
* Proteus mirabilis*	1 (0.9)	0	0	0	0	1 (0.4)
* *Non-fermentative Gram-negative bacilli	18 (15.9)	14 (20)	8 (17.8)	0	2 (13.3)	42 (16)
* Pseudomonas aeruginosa*	5 (4.4)	9 (12.9)	4 (8.9)	0	1 (6.7)	19 (7.3)
* Acinetobacter baumannii*	6 (5.3)	4 (5.7)	1 (2.2)	0	0	11 (4.2)
* Acinetobacter nosocomialis*	4 (3.5)	0	1 (2.2)	0	0	5 (1.9)
* Aeromonas hydrophila*	0	0	1 (2.2)	0	1 (6.7)	2 (0.8)
* Stenotrophomonas maltophilia*	1 (0.9)	1 (1.4)	0	0	0	2 (0.8)
* Acinetobacter xylosoxidans*	1 (0.9)	0	0	0	0	1 (0.4)
* Burkholderia cepacia*	1 (0.9)	0	1 (2.2)	0	0	2 (0.8)
* Candida* spp.	6 (5.3)	0	2 (4.4)	2 (10.5)	0	10 (3.8)
* *Other	1 (0.9)	1 (1.4)	0	0	0	2 (0.8)
* Clostridium*	1 (0.9)	0	0	0	0	1 (0.4)
* Haemophilus influenzae*	0	1 (1.4)	0	0	0	1 (0.4)
Total	113 (100)	70 (100)	45 (100)	19 (100)	15 (100)	262 (100)

In patients with ICU-BSI caused by a bacterial microorganism, initial antibiotic therapy was appropriate in 65.1% of cases. There was no difference in the rate of appropriate initial antibiotic therapy according to the causative microorganism or to its resistance profile.

### Intensive care unit–acquired bloodstream infection in ESBL-PE carriers.

Epidemiological and clinical data of patients with ICU-BSIs caused by ESBL and non–ESBL-PE are reported in Supplemental Appendix A. The causative microorganism of the first episode of ICU-BSI was ESBL-PE in 37 patients (16.6%). In patients with previous ESBL-PE carriage, the causative microorganism of the first episode of ICU-BSI was ESBL-PE in 29.8% of cases. In multivariate analysis, factors independently associated with ESBL-PE as the causative microorganism of ICU-BSI were ESBL-PE carriage before ICU-BSI (OR: 7.273; 95% CI: 2.876–18.392; *P* < 0.000) and prior exposure to fluoroquinolones (OR: 4.327; 95% CI: 1.120–16.728; *P* = 0.034).

In ESBL-PE carriers (before the ICU-BSI), 24 patients (40.7%) developed ICU-BSI caused by ESBL-PE. The Ss of ESBL-PE carriage to predict ESBL-PE as the causative microorganism of ICU-BSI was 64.9% and the Sp was 81.2%. The PPV was 40.7% and the NPV was 92.1%. Predictive values of MDR-B carriage to predict ICU-BSI caused by the same organism are reported in Table 6.

**Table 6:** Predictive value of multidrug-resistant bacteria carriage to predict ICU-BSI caused by the same organism

Carriage before ICU-BSI	Total	Nb ICU-BSI*	Sensitivity	Specificity	Positive predictive value	Negative predictive value
Multidrug-resistant bacteria carriage	69	45	0.67	0.79	0.44	0.9
ESBL-PE carriage	57	37	0.65	0.81	0.41	0.92
ESBL-producing *Escherichia coli* carriage	18	1	1	0.92	0.06	1
ESBL-producing *Klebsiella pneumoniae* carriage	64	29	0.9	0.8	0.41	0.98
ESBL-producing *Enterobacter* spp. carriage	27	10	0.3	0.89	0.11	0.96

ESBL-PE = extended-spectrum beta-lactamase–producing Enterobacteriaceae; ICU-BSI = intensive care unit–acquired bloodstream infections.

* Nb of ICU-BSI caused by the same microorganism.

### Outcome.

Mortality rate in ICU was 25.6% in the general population. It was 21.5% in ESBL-PE carriers, 25% in ESBL-PE carriers with ICU-BSI caused by ESBL-PE versus 20% in ESBL-PE carriers with ICU-BSI caused by non ESBL-PE (*P* = ns).

Mortality at 28 days was 20.6% in the general population. It was 25.6% in patients with ICU-BSI caused by ESBL-PE versus 19.6% in patients with ICU-BSI caused by non–ESBL-PE (*P* = ns). Epidemiological and clinical data of patients according to the 28-day mortality are reported in Supplemental Appendix B.

The median ICU length of stay was 26 days (IQR: 15–49). It was 37 days (IQR: 18–57) in patients with ICU-BSI caused by ESBL-PE versus 24 days (IQR: 15–48) in patients with ICU-BSI caused by non–ESBL-PE (*P* = ns).

In multivariable analysis, factors independently associated with mortality at day 28 from the occurrence of ICU-BSI were trauma at admission (OR: 0.346; 95% CI: 0.134–0.894; *P* = 0.028) and septic shock associated with ICU-BSI (OR: 3.317; 95% CI: 1.561–7.050; *P* = 0.002).

Comparison of the mortality at day 28 according to the causative organism with the mortality recorded in cases of ICU-BSI caused by methicillin-susceptible *Staphylococcus aureus* showed no statistical difference between organisms (Table 7).

**Table 7:** Mortality at day 28 according to the causative organism

Organism	Mortality (%)	Odds ratio	Minimum	Maximum	*P*-value
Methicillin-susceptible *Staphylococcus aureus*	18.2	Reference	–	–	–
Enterobacteriaceae	17.3	0.255	0.341	4.614	0.732
*Klebsiella pneumoniae*	9.2	0.245	0.114	1.772	0.245
*Enterobacter* spp.	14.9	0.788	0.204	3.033	0.728
*Escherichia coli*	37.5	2.700	0.613	11.892	0.182
Extended-spectrum beta-lactamase–producing Enterobacteriaceae	21.6	1.241	0.326	4.725	0.751
Non-fermentative Gram-negative bacilli	12.2	0.644	0.153	2.715	0.447
*Pseudomonas aeruginosa*	10.5	0.529	0.086	3.275	0.489
*Acinetobacter baumannii*	9.1	0.450	0.044	4.596	0.492
*Candida* spp.	40.0	3.000	0.567	15.867	0.186
Coagulase-negative staphylococci	33.3	2.250	0.490	10.341	0.292

## DISCUSSION

Our study shows that ICU-BSI is a frequent complication in ICU patients and that it was primitive in most cases. The isolated microorganisms were dominated by Enterobacteriaceae and an ESBL-PE was isolated in one-fourth of cases. Factors significantly associated with ICU-BSI caused by ESBL-PE were ESBL-PE carriage and exposure to fluoroquinolones before ICU-BSI. The mortality remains elevated, and associated prognosis factors were non-traumatic at admission and septic shock on the day of the ICU-BSI. Analysis of mortality according to the causative organism, when compared with the mortality recorded in cases of ICU-BSI caused by methicillin-susceptible *S. aureus*, showed no difference between organisms.

In the published literature, ICU-BSI caused complications in 2–7% of admissions, with an incidence rate ranging between four and nine per 1,000 patient days at risk.^2,3,7,24^ The median time to develop ICU-BSI is 7–18 days from admission.^2,6^ It depended on the causative bacteria, with 13 days for *E. coli*, 37 days for *K. pneumoniae*, and 11 days for *Proteus mirabilis*.^8,24^ In our study, ICU-BSIs complicated 9.5% of admissions, giving a density incidence of 10.3 ICU-BSI/1,000 days of hospitalization. This high rate can be explained by the tropical location of our unit. The median time to the first positive blood culture was 9 days, similar to the rates reported in the literature.^2,6^

Bloodstream infections may be the consequence of the bloodstream diffusion of microorganisms from a localized infection (secondary BSI) or may be the only identifiable infectious process (primary BSI).^11^ The primary ICU-BSIs are the most frequent (23–33.5% of cases).^6,24–26^ For secondary ICU-BSIs, the major reported causes are catheter-related BSIs in most studies (21–30%), lung infection or ventilator-associated pneumonia (VAP) (15–21%), biliary and urinary tract infections (14–45%), surgical wounds, peritonitis, or soft tissue infection.^6,24–26^ In our study, we found a high level of primary ICU-BSIs (44%). For secondary ICU-BSIs, the major causes were VAP (25.6%) and catheter-related infection (17.5%). These rates are concordant with those reported in the literature. A deeper analysis of the characteristics of primary ICU-BSIs is needed to create a better understanding of its predisposing factors and its preventive measures.

In the EUROBACT study, 57.6% of microorganisms responsible for ICU-BSI were Gram negative and 33.4% were Gram positive.^6^ *Candida* spp. is isolated in 8–15% of cases of ICU-BSI.^2,6^ Prowle et al.^2^ found that the main causative microorganisms of ICU-BSI were Gram-negative bacilli (28.2%), *S. aureus* (26.7%), coagulase-negative staphylococci (24.3%), enterococci (17.0%), and *Candida* spp. (15.5%). Corona et al.^20^ found that the main causative microorganisms of ICU-BSI were Gram-negative bacilli (37.3%), coagulase-negative staphylococci (29.6%), *S. aureus* (23.6%), enterococci (11.4%), and *Candida* spp. (6.5%). *Candida* spp. plays a major role in ICU-BSI, accounting for 6–15% of cases.^6^ In a fungemia subgroup of the EUROBACT study, *Candida albicans* was the most frequent fungus isolated (57.1%), followed by *Candida glabrata* (15.3%) and *Candida parapsilosis* (10.2%).^28^ In our study, the microorganism recovered was an Enterobacteriaceae in 68% of patients and a Gram-positive cocci in 26% of patients. It was an ESBL producer in 37 cases (24.5%). *Candida* spp. was recovered in 10 cases (4.9%). The causative microorganism was ESBL-PE in 17% of patients. In patients with previous ESBL-PE carriage, the causative microorganism of the first episode of ICU-BSI was ESBL-PE in 29.8% of cases. In multivariable analysis, factors independently associated with ESBL-PE as the causative microorganism of ICU-BSI were ESBL-PE carriage before ICU-BSI (OR: 7.273; 95% CI: 2.876–18.392; *P* < 0.000) and prior exposure to fluoroquinolones (OR: 4.327; 95% CI: 1.120–16.728; *P* = 0.034). These results are important to identify patients with suspected ESBL-PE in cases of ICU-BSI and to guide initial empiric antibiotic therapy.

Patients with ICU-BSI have a longer ICU or hospital stay.^2,24^ In addition, ICU-BSI is independently associated with a higher mortality rate.^3,5,6,24,29^ Prowle et al.^2^ found that the mortality rate was 2-fold higher in patients with BSI. However, only 5% of the deaths in his study could be attributed to ICU-BSI, equivalent to an absolute decrease in survival of 1% of the total population.^2^ When analyzed by microbiological classification, *Candida* spp., *S. aureus*, and Gram-negative bacilli infections were independently associated with increased risk of death. In a subgroup analysis, intravascular catheter-associated BSIs remained associated with significant risk of death (hazard ratio: 2.64; 95% CI: 1.44–4.83; *P* = 0.002).^2^ In addition, source control of ICU-BSI is shown to be independently related to the outcome.^5^ Some sources are associated with a higher fatality rate, for example, respiratory,^30^ catheter-associated BSI,^2^ unknown,^24^ or abdominal sources.^3,6^ On the other hand, some authors found that BSI due to catheter-related infection did not increase the risk of death.^24^ In addition, some authors suggest that the virulence of the microorganism rather than the source of infection may be more important in determining outcomes and that prevention of these infections (predominantly *S. aureus*, Gram-negative bacilli, and *Candida* spp.) is an important therapeutic goal.^2^ Indeed, compared with *S. aureus* and adjusted by age, gender, and type of ICU, *Stenotrophomonas maltophilia* was associated with significantly higher ICU mortality (OR 1.71), followed by enterococci (OR 1.20), *E. coli* (OR 1.24), *C. albicans* (OR 1.37), *non-albicans Candida* spp. (OR 1.49), and *Pseudomonas aeruginosa* (OR 1.49).^31^ Other factors are reported to be associated with 28-day mortality such as older patients, chronic respiratory disease or immune deficiency, septic shock or higher SOFA score or cardiac diseases, organ dysfunction within 2 days before ICU-BSI, transfer from another ward, nutrition, intravenous or urinary tract catheters within a week before, and/or do-not-resuscitation order.^6,24^ In our study, mortality at 28 days was 20.6% in the general population and 15.7% in patients with ESBL-PE carriage. It was 19.4% in ESBL-PE carriers with ICU-BSI caused by ESBL-PE versus 14.1% in ESBL-PE carriers with ICU-BSI caused by non–ESBL-PE (nonsignificant). Analysis of the impact of causative microorganism of ICU-BSI compared with *S. aureus* at 28-day mortality showed no difference between organisms. In multivariable analysis, factors independently associated with mortality at day 28 from the occurrence of ICU-BSI were of a traumatic category at admission (OR: 0.346; 95% CI: 0.134–0.894; *P* = 0.028), and septic shock associated with ICU-BSI (OR: 3.317; 95% CI: 1.561–7.050; *P* = 0.002). Indeed, septic shock is a severe condition associated with a high mortality rate reaching 40% in some cases.^21^ Regarding patients with trauma at admission, they are typically younger and in good health, placing them at a lower risk of mortality in ICU.

In cases of ICU-BSI, the impact of early appropriate antibiotic therapy on outcomes is controversial. Kumar et al.^32^ found that inadequate therapy within 6 hours after onset of hypotension was associated with more than a 9-fold increase in the risk of death in patients with septic shock and documented BSIs. However, Vallés et al.^33^ found that appropriate antibiotic therapy (given in the 24 hours after the availability of blood sample test results) had no influence on mortality. Corona et al.^20^ investigated the delay from the first positive blood culture sampling to the first day of effective antibiotic therapy against the microorganisms isolated. The adjusted ORs for death were 1.34, 1.75, and 0.97 at 1, 2, and 3 days, respectively (nonsignificant), indicating the absence of the effect on mortality. Similar results were obtained in the EUROBACT study^6^ where very early treatment (< 1 day after the first positive blood culture taken) was not associated with a decrease in the risk of death as compared with less than 2 days and less than 5 days. One of the limitations in comparing these results is that the definition of adequate or appropriate antimicrobial therapy in ICU patients varies between studies.^20,33,34^ In our study, analysis of patients with ICU-BSI caused by a bacterial microorganism showed an appropriateness level of initial antibiotic therapy at 65.1%. Comparison of patients with and without appropriate initial antibiotic therapy did not find any difference in the outcome. This result is similar to others reported in the literature but should be interpreted with caution in the absence of adjustment on the severity at the moment of the diagnosis of ICU-BSI. Clinicians have to find the balance between providing an early adequate empiric coverage and a rational use of broad-spectrum antimicrobials. Indeed, focusing solely on the goal of providing a broad-spectrum antibiotic can be a driver for overuse of antimicrobials, which is one of the main causes of the increase in drug resistance.

Our study has several limitations. First, it is a retrospective monocentric work. But our ICU is the referral center (being the only ICU in the region) for a large population coming from the costal side of the Amazonian region (French Guiana, Brazil, and Suriname). Second, the sample size is not large enough to adjust for confounding variables. In addition, we did not study some time-dependent variables such as severity of illness on the day of the diagnosis of ICU-BSI.

## CONCLUSION

Intensive care unit–acquired bloodstream infection is a major complication in ICU patients. The main sources are VAP, catheter-associated infection, plus some as yet unknown infection. The isolated microorganisms were dominated by Enterobacteriaceae with a high rate of ESBL-PE. Extended-spectrum beta-lactamase-PE carriage and exposure to fluoroquinolones before ICU-BSI are significantly associated with ICU-BSI caused by ESBL-PE. Mortality remains high and associated with the non-traumatic category at admission and septic shock on the day of the ICU-BSI. Further studies with deeper analysis and adjustment on confounding variables are needed to search for pertinent associated factors affecting the outcome of ICU-BSI allowing rapid detection of at-risk patients and targeted preventive measures.

## Supplemental appendixes

Supplemental materials
