# Supplementary material for: Epidemiology and Prognosis of Intensive Care Unit–Acquired Bloodstream Infection
Source: Am J Trop Med Hyg. 2020 Apr 20;103(1):508–14. doi: 10.4269/ajtmh.19-0877 (PMC7356483; doi:10.4269/ajtmh.19-0877)
Supplement: Supplementary file 1 [file tpmd190877.SD1.pdf]

Supplemental Appendix A: Epidemiological and clinical parameters and outcome of patients with USI-BSI caused by ESBL-PE and non ESBL-PE

**Appendix A (part 1)**	**ICU-BSI caused by BLSE-PE**		**ICU-BSI caused by non BLSE-PE**		***p***
**Parameter**	**Nb**	**Result**	**Nb**	**Result**	
Age, years	39	46 (32 - 67)	184	49 (36 - 60)	0.620
Male gender	39	20 (51.3%)	184	130 (70.7%)	0.024
BMI	34	25 (24 - 34)	157	25 (22 - 29)	0.022
SAPS II	39	57 (42 - 75)	180	50 (39 - 63)	0.045
Type of admission					
Traumatic	39	4 (10.3%)	184	64 (34.8%)	0.004
Medical past	39	25 (64.1%)	184	104 (56.5%)	0.589
Arterial hypertension	39	11 (28.2%)	184	60 (32.6%)	0.763
Diabetes mellitus	39	7 (17.9%)	184	22 (12%)	0.242
Cancer	39	4 (10.3%)	184	5 (2.7%)	0.022
Immunodeficiency	39	13 (33.3%)	184	27 (14.7%)	0.336
Chronic renal failure	39	3 (7.7%)	184	8 (4.3%)	0.329
Chronic respiratory failure	39	1 (2.6%)	184	3 (1.6%)	0.648
Sickle cell disease	39	4 (10.3%)	184	6 (3.3%)	0.244
Organ failure at admission	39	3 (2 - 4)	184	3 (2 - 3)	0.031
Hemodynamic	39	31 (79.5%)	184	112 (60.9%)	0.048
Respiratory	39	30 (76.9%)	184	116 (63%)	0.029
Neurologic	39	26 (66.7%)	184	123 (66.8%)	0.915
Kidney	39	17 (43.6%)	184	57 (31%)	0.071
Liver	39	10 (25.6%)	184	18 (9.8%)	0.018
Hematologic	39	12 (30.8%)	184	37 (20.1%)	0.092
Mechanical ventilation	39	37 (94.9%)	184	170 (92.4%)	0.648
Time from admission to MV	37	0 (0 - 0)	170	0 (0 - 0)	0.908
Duration of MV.days	37	26 (14 - 42)	170	20 (12 - 31)	0.339
Ventilator Associated Pneumoniae	37	14 (37.8%)	170	83 (48.8%)	0.372
Duration of MV without VAP. days	14	11 (8 - 17)	83	7 (4 - 14)	0.595
Tracheostomy	37	5 (13.5%)	170	32 (18.8%)	0.543
Time from admission to tracheostomy	5	33 (33 - 40)	33	29 (23 - 42)	0.912
Unscheduled detubation	37	1 (2.7%)	170	6 (3.5%)	0.851
Renal replacement therapy	39	10 (25.6%)	184	33 (17.9%)	0.191
Time from admission to RRT	10	1 (0 - 3)	33	2 (0 - 8)	0.437
Active infection at admission	39	28 (71.8%)	184	89 (48.4%)	0.018
BSI at admission	28	7 (25%)	89	16 (18%)	0.950
Antibiotics at admission	39	35 (89.7%)	184	119 (64.7%)	0.004
Amoxicillin clavulanate	39	9 (23.1%)	184	61 (33.2%)	0.311
Aminoglycosides	39	10 (25.6%)	184	21 (11.4%)	0.012
Piperacillin-Tazobactam	39	13 (33.3%)	184	22 (12%)	0.000
3rd generation cephalosporins	39	8 (20.5%)	184	26 (14.1%)	0.238
Imipenem	39	3 (7.7%)	184	7 (3.8%)	0.767
Fluoro-quinolones	39	9 (23.1%)	184	9 (4.9%)	0.000

**Appendix A (part 2)**	**ICU-BSI caused by BLSE-PE**		**ICU-BSI caused by non BLSE-PE**		***p***
**Parameter**	**Nb**	**Result**	**Nb**	**Result**	
Metronidazole	39	1 (2.6%)	184	2 (1.1%)	0.433
Central venous catheter (CVC)	39	38 (97.4%)	184	178 (96.7%)	0.868
CVC related Infection	39	12 (30.8%)	184	38 (20.7%)	0.110
Overall duration of CVC	39	27 (15 - 47)	184	19 (11 - 32)	0.076
Duration of CVC without infection	12	21 (11 - 29)	38	16 (9 - 26)	0.822
Arterial catheter (AC)	39	37 (94.9%)	184	169 (91.8%)	0.217
AC related Infection	39	4 (10.3%)	184	29 (15.8%)	0.455
Overall duration of AC	39	17 (13 - 26)	184	14 (9 - 21)	0.028
Duration of AC without infection	3	14 (14 - 19)	26	9 (8 - 15)	0.330
Multi-drug Resistant Bacteria carriage	39	38 (97.4%)	184	103 (56%)	0.000
ESBL-PE carriage	39	38 (97.4%)	184	83 (45.1%)	0.000
ESBL-PE carriage at admission	39	11 (28.2%)	184	17 (9.2%)	0.018
ICU-BSI microbiology					
ICU-BSI caused by one organism	39	34 (87.2%)	184	151 (82.1%)	0.802
Non-fermenting organism	39	2 (5.1%)	184	40 (21.7%)	0.030
Enterobacteriaceae	39	39 (100%)	184	86 (46.7%)	0.000
ESBL-PE	39	39 (100%)	184	0 (0%)	0.000
Candida Spp	39	0 (0%)	184	10 (5.4%)	0.129
Methicillin sensitive S*taphylococcus aureus*	39	0 (0%)	184	22 (12%)	0.028
Coagulase negative staphylococcus	39	0 (0%)	184	13 (7.1%)	0.074
Septic shock	39	9 (23.1%)	184	37 (20.1%)	0.882
Appropriate ATBth within 24h	39	24 (61.5%)	184	127 (69%)	0.826
ESBL-PE carriage prior to ICU-BSI	39	26 (66.7%)	184	33 (17.9%)	0.000
ATB exposure prior to ICU-BSI					
Amoxicillin clavulanate	39	8 (20.5%)	184	57 (31%)	0.270
Aminoglycosides	39	17 (43.6%)	184	56 (30.4%)	0.061
Piperacillin-Tazobactam	39	12 (30.8%)	184	44 (23.9%)	0.261
3rd generation cephalosporins	39	14 (35.9%)	184	34 (18.5%)	0.008
Imipenem	39	6 (15.4%)	184	18 (9.8%)	0.554
Fluoroquinolones	39	8 (20.5%)	184	11 (6%)	0.002
Metronidazole	39	1 (2.6%)	184	5 (2.7%)	0.996
Outcome					
ICU LOS, days	39	37 (18 - 57)	184	24 (15 - 48)	0.731
Length of stay greater than 48 hours	39	39 (100%)	184	183 (99.5%)	0.655
Time from admission to BSI	39	12 (8 - 18.5)	184	8 (5 - 14)	0.018
Delay between ICU-BSI and ICU discharge	39	20 (8 - 35)	184	14 (6 - 35)	0.953
Death	39	12 (30.8%)	184	45 (24.5%)	0.823
28-day mortality	39	10 (25.6%)	184	36 (19.6%)	0.870

Supplemental Appendix B: Epidemiological and clinical parameters of patients with USI-BSI according to the 28-day mortality

**Appendix B (Part 1)**	**Dead at 28 day**		**Alive at 28 day**		***p***
**Parameter**	**Nb**	**Result**	**Nb**	**Result**	
Age, years	46	54 (43 - 64)	177	47 (33 - 60)	0.026
Male gender	46	31 (67.4%)	177	119 (67.2%)	0.984
BMI	40	24 (21 - 31)	151	25 (22 - 30)	0.398
SAPS II	44	62 (46 - 76)	175	49 (39 - 61)	0.005
Type of admission					
Traumatic	46	6 (13%)	177	62 (35%)	0.004
Medical past	46	32 (69.6%)	177	97 (54.8%)	0.183
Arterial hypertension	46	18 (39.1%)	177	53 (29.9%)	0.223
Diabetes mellitus	46	7 (15.2%)	177	22 (12.4%)	0.616
Cancer	46	0 (0%)	177	9 (5.1%)	0.118
Immunodeficiency	46	12 (26.1%)	177	28 (15.8%)	0.049
Chronic renal failure	46	6 (13%)	177	5 (2.8%)	0.004
Chronic respiratory failure	46	1 (2.2%)	177	3 (1.7%)	0.827
Sickle cell disease	46	2 (4.3%)	177	8 (4.5%)	0.960
Organ failure at admission	46	3 (2 - 4)	177	2 (2 - 3)	0.087
Hemodynamic	46	36 (78.3%)	177	107 (60.5%)	0.025
Respiratory	46	33 (71.7%)	177	113 (63.8%)	0.316
Neurologic	46	31 (67.4%)	177	118 (66.7%)	0.926
Kidney	46	22 (47.8%)	177	52 (29.4%)	0.018
Liver	46	8 (17.4%)	177	20 (11.3%)	0.267
Hematologic	46	13 (28.3%)	177	36 (20.3%)	0.248
Mechanical ventilation	46	44 (95.7%)	177	163 (92.1%)	0.404
Time from admission to MV	44	0 (0 - 0)	163	0 (0 - 0)	0.193
Duration of MV, days	44	14.5 (11 - 24)	163	23 (14 - 39)	0.008
Ventilator Associated Pneumoniae	44	16 (36.4%)	163	81 (49.7%)	0.116
Duration of MV without VAP, days	16	8 (4 - 14)	81	7 (5 - 16)	0.465
Tracheostomy	44	1 (2.3%)	163	36 (22.1%)	0.002
Time from admission to tracheostomy	1	19 (19 - 19)	37	30 (24 - 40)	0.336
Unscheduled detubation	44	2 (4.5%)	163	5 (3.1%)	0.630
Renal replacement therapy	46	16 (34.8%)	177	27 (15.3%)	0.003
Time from admission to RRT	16	2 (0 - 9)	27	1 (0 - 4)	0.271
Active infection at admission	46	24 (52.2%)	177	93 (52.5%)	0.964
BSI at admission	24	8 (33.3%)	93	15 (16.1%)	0.059
Antibiotics at admission	46	28 (60.9%)	177	126 (71.2%)	0.177
Amoxicillin clavulanate	46	8 (17.4%)	177	62 (35%)	0.022
Aminoglycosides	46	7 (15.2%)	177	24 (13.6%)	0.772
Piperacillin-Tazobactam	46	9 (19.6%)	177	26 (14.7%)	0.418
3rd generation cephalosporins	46	6 (13%)	177	28 (15.8%)	0.641
Imipenem	46	3 (6.5%)	177	7 (4%)	0.454
Fluoro-quinolones	46	2 (4.3%)	177	16 (9%)	0.298
Metronidazole	46	0 (0%)	177	3 (1.7%)	0.374

**Appendix B (Part 2)**	**Dead at 28 day**		**Alive at 28 day**		***p***
**Parameter**	**Nb**	**Result**	**Nb**	**Result**	
Central venous catheter (CVC)	46	45 (97.8%)	177	171 (96.6%)	0.673
CVC related Infection	46	11 (23.9%)	177	39 (22%)	0.785
Overall duration of CVC	46	16 (10 - 28)	177	21 (13 - 41)	0.023
Duration of CVC without infection	11	9 (7 - 16)	39	18 (13 - 29)	0.044
Arterial catheter (AC)	46	42 (91.3%)	177	164 (92.7%)	0.758
AC related Infection	46	5 (10.9%)	177	28 (15.8%)	0.400
Overall duration of AC	46	13 (8 - 19)	177	16 (10 - 23)	0.042
Duration of AC without infection	4	9 (8 - 10)	25	12 (8 - 18)	0.367
Multi-drug Resistant Bacteria carriage	46	23 (50%)	177	118 (66.7%)	0.037
ESBL-PE carriage	46	19 (41.3%)	177	102 (57.6%)	0.048
ESBL-PE carriage at admission	46	7 (15.2%)	177	21 (11.9%)	0.541
ICU-BSI microbiology					
ICU-BSI caused by one organism	46	43 (93.5%)	177	141 (79.7%)	0.028
Non-fermenting organism	46	6 (13%)	177	34 (19.2%)	0.332
Enterobacteriaceae	46	22 (47.8%)	177	111 (62.7%)	0.067
ESBL-PE	46	8 (17.4%)	177	29 (16.4%)	0.870
Candida Spp	46	4 (8.7%)	177	7 (4%)	0.186
Methicillin sensitive S*taphylococcus aureus*	46	4 (8.7%)	177	18 (10.2%)	0.765
Coagulase negative staphylococcus	46	5 (10.9%)	177	10 (5.6%)	0.208
Septic shock	46	19 (41.3%)	177	27 (15.3%)	0.000
Appropriate ATBth within 24h	46	30 (65.2%)	177	121 (68.4%)	0.685
ESBL-PE carriage prior to ICU-BSI	46	14 (30.4%)	177	45 (25.4%)	0.492
ATB exposure prior to ICU-BSI	46	29 (63%)	177	130 (73.4%)	0.165
Amoxicillin clavulanate	46	9 (19.6%)	177	56 (31.6%)	0.108
Aminoglycosides	46	13 (28.3%)	177	60 (33.9%)	0.468
Piperacillin-Tazobactam	46	8 (17.4%)	177	48 (27.1%)	0.175
3rd generation cephalosporins	46	7 (15.2%)	177	41 (23.2%)	0.243
Imipenem	46	7 (15.2%)	177	17 (9.6%)	0.274
Fluoro-quinolones	46	3 (6.5%)	177	16 (9%)	0.586
Metronidazole	46	1 (2.2%)	177	5 (2.8%)	0.808
Outcome					
ICU LOS, days	46	17 (11 - 24)	177	32 (17 - 58)	0.002
Length of stay greater than 48 hours	46	46 (100%)	177	176 (99.4%)	0.609
Time from admission to BSI	46	8 (5 - 16)	177	9 (5 - 15)	0.607
Delay between ICU-BSI and ICU discharge	46	6 (2 - 14)	177	19 (8 - 39)	0.002
Death	46	46 (100%)	177	11 (6.2%)	0.000
